# Comparative evaluation of the activity of novel β-lactam antibiotics against difficult-to-treat *Pseudomonas aeruginosa* clinical isolates

**DOI:** 10.1093/jacamr/dlag138

**Published:** 2026-07-16

**Authors:** Harry Mystakelis, Ryan K Shields, Amy J Mathers, Yehudit Bergman, Jose M Munita, Sara E Cosgrove, Patricia J Simner, Pranita D Tamma

**Affiliations:** Department of Pediatrics, Johns Hopkins University School of Medicine, Baltimore, MD, USA; Department of Medicine, University of Pittsburgh, Pittsburgh, PA, USA; Department of Medicine, University of Virginia, Charlottesville, VA, USA; Department of Pediatrics, University of Pennsylvania Perelman School of Medicine, Philadelphia, PA, USA; Facultad de Medicina Clínica Alemana, Universidad del Desarrollo, Instituto de Ciencias e Innovación en Medicina, Santiago, Chile; Department of Medicine, Johns Hopkins University School of Medicine, Baltimore, MD, USA; Department of Pathology, Mayo Clinic, Rochester, MN, USA; Department of Pediatrics, University of Pennsylvania Perelman School of Medicine, Philadelphia, PA, USA

## Abstract

**Objectives:**

This study aimed to characterize susceptibility profiles of both currently approved and investigational β-lactam agents against difficult-to-treat resistant *Pseudomonas aeruginosa* (DTR *P. aeruginosa*) clinical isolates, including carbapenemase-producing strains.

**Patients and methods:**

A total of 502 consecutive DTR *P. aeruginosa* clinical isolates were collected from unique hospitalized patients between 2022 and 2024 across three tertiary care centres. Isolates originated from bloodstream, respiratory, urinary and intra-abdominal infections. Minimum inhibitory concentrations (MICs) for seven β-lactam agents were determined using triplicate reference broth microdilution. Susceptibility interpretations were based on CLSI criteria or, where applicable, investigational breakpoints. Whole-genome sequencing was performed to detect β-lactamase genes.

**Results:**

Among the 502 isolates, cefepime-zidebactam exhibited the highest *in vitro* activity (100% susceptibility), followed by cefiderocol-xeruborbactam (98%), cefiderocol (95%), ceftolozane-tazobactam (90%), cefepime-taniborbactam (86%), ceftazidime-avibactam (85%), and imipenem-relebactam (41%). Notably, only 35% of isolates were susceptible to all seven agents. Carbapenemase genes were identified in 4% of isolates, including *bla*_GES_ (*n* = 13), *bla*_VIM_ (*n* = 7), *bla*_KPC_ (*n* = 1) and *bla*_IMP_ (*n* = 1). Among carbapenemase-producing isolates, cefepime-zidebactam retained activity against all isolates (100%), exceeding that of other approved β-lactam/β-lactamase inhibitor combinations (0%–24% susceptible), cefiderocol (76%), cefiderocol-xeruborbactam (86%) and cefepime-taniborbactam (81%).

**Conclusions:**

While currently approved β-lactam agents retained activity against many DTR *P. aeruginosa* isolates, their performance was markedly compromised in the presence of carbapenemases, with the notable exceptions of cefepime-zidebactam and cefiderocol. Investigational β-lactam/β-lactamase inhibitor combinations exhibited consistently robust activity across resistance phenotypes, including carbapenemase-producing isolates, representing promising options for DTR *P. aeruginosa* infections.

## Introduction

Difficult-to-treat (DTR) *Pseudomonas aeruginosa*—defined as *P. aeruginosa* isolates not susceptible to all traditional β-lactams (e.g. cefepime, meropenem) and fluoroquinolones (e.g. ciprofloxacin)—represent an escalating international health threat.^1^  *P. aeruginosa* possesses an extraordinary capacity to acquire and upregulate diverse antimicrobial resistance determinants, limiting therapeutic options and contributing to mortality exceeding 30%.^2^

Resistance to β-lactams by *P. aeruginosa* commonly arises through multiple, often concurrent mechanisms. These include loss or downregulation of porins (e.g. OprD), overexpression of multidrug efflux pumps (e.g. MexAB-OprM), hyperproduction or structural modifications of *Pseudomonas*-derived cephalosporinases (PDCs), acquisition of carbapenemases (e.g. VIM enzymes) and target-site modifications that reduce β-lactam binding affinity (e.g. altered PBP3).^3^

Since 2015, several β-lactam agents with enhanced activity against DTR *P. aeruginosa* have become available in the USA, including cefiderocol, ceftazidime-avibactam, ceftolozane-tazobactam and imipenem-relebactam.^4^ More recently, cefepime-zidebactam received US FDA approval in May 2026, while cefepime-taniborbactam and cefiderocol-xeruborbactam remain investigational.^5^ These newer agents incorporate novel β-lactamase inhibitors engineered to overcome resistance mechanisms commonly encountered in DTR phenotypes. However, given continued shared structural features and overlapping targets, cross-resistance remains plausible between FDA-approved agents and investigational β-lactam-β-lactamase inhibitors (BL-BLIs).

In anticipation of the clinical availability of newer agents with activity against DTR *P. aeruginosa*, we sought to investigate the likelihood of resistance and cross-resistance in a multicentre cohort of DTR *P. aeruginosa* clinical isolates from the mid-Atlantic USA.

## Methods

The cohort comprised 502 DTR *P. aeruginosa* clinical isolates (i.e. resistant to all traditional β-lactams and fluoroquinolones) from unique, consecutive patients hospitalized at three mid-Atlantic hospitals (The Johns Hopkins Hospital, The University of Pittsburgh Medical Center and The University of Virginia) between 2022 and 2024. Eligible isolates, defined as the first DTR isolate per patient during the study, were obtained from clinically significant infections involving the bloodstream, respiratory tract, urine or intra-abdominal fluid, as determined by an infectious diseases specialist. Isolates from patients with cystic fibrosis were excluded due to challenges in accurate minimum inhibitory concentration (MIC) determination from mucoid specimens. Baseline patient data at the time of culture collection included demographics, travel history, comorbidities, severity of illness and infection source.

Initial species identification and antimicrobial susceptibility testing (AST) were performed at local clinical microbiology laboratories using matrix-assisted laser desorption ionization–time of flight mass spectrometry (Bruker Daltonics) and either the BD Phoenix^®^ (Becton Dickinson), MicroScan WalkAway (Beckman Coulter) or VITEK^®^ 2 (bioMérieux) systems. Isolates with meropenem MICs ≥8 mg/L were stored at −80°C in glycerol and subsequently batch-shipped to The Johns Hopkins Hospital for centralized testing.

Upon receipt, isolates were subcultured twice on tryptic soy agar with 5% sheep blood. Species identification was confirmed by MALDI-TOF MS. Reference broth microdilution (BMD) panels (Thermo Fisher Scientific, USA) with cation-adjusted Mueller–Hinton broth (CA-MHB; BD Difco base) were prepared to determine MICs for seven β-lactam agents. Iron-depleted CA-MHB was used for cefiderocol and cefiderocol-xeruborbactam. β-lactam concentrations ranged from ≤0.125 to ≥256 mg/L and all inhibitors were fixed at 4 g/L, with the exception of cefepime-zidebactam which was tested in a 1:1 ratio.^6^ Testing was conducted in technical triplicate to derive modal MIC values. Quality control strains were selected in accordance with Clinical and Laboratory Standards Institute (CLSI) M100 guidelines and performed on each day of testing.^6^

The following interpretive criteria were applied to determine susceptibility: cefepime-taniborbactam ≤8/4 mg/L (CLSI cefepime breakpoint), cefepime-zidebactam ≤64/64 mg/L (CLSI investigational breakpoint), cefiderocol ≤4 mg/L (CLSI breakpoint), cefiderocol-xeruborbactam ≤4/4 mg/L (CLSI cefiderocol breakpoint), ceftazidime-avibactam ≤8/4 mg/L (CLSI breakpoint), ceftolozane-tazobactam ≤4/4 mg/L (CLSI breakpoint), imipenem-relebactam ≤2/4 mg/L (CLSI breakpoint).^6^ Percent susceptibilities, MIC_50_ and MIC_90_ were calculated for each antibiotic.

All isolates underwent genomic DNA extraction using the DNeasy PowerSoil Pro Kit (QIAGEN, Germantown, MD, USA). Short-read whole-genome sequencing was performed on the Illumina NextSeq 2000 platform to characterize β-lactamase genes. AREScloud (Ares Genetics, Vienna, Austria) was used for genome assembly, organism identification, sequence type, plasmid type and antimicrobial resistance gene detection. Acknowledging the variability in carbapenem hydrolysis across *bla*_GES_ alleles,^7^ they were categorized as carbapenemase genes for this study.

## Results

Baseline characteristics of the 502 patients contributing eligible isolates are summarized in Table 1. The median age was 62 years (IQR 52–71). Overall, 297 (59%) of patients were admitted to the intensive care unit at the time of culture collection and 127 (25%) met criteria for severe immunocompromise. Pneumonia predominated (*n* = 307, 61%), followed by intra-abdominal (*n* = 88, 18%), urinary (*n* = 77, 15%) and bloodstream infections (*n* = 30, 6%).

**Table 1.** Baseline characteristics of 502 unique patients with *Pseudomonas aeruginosa* isolates exhibiting difficult-to-treat resistance

Baseline characteristics	Number (percent)
Median age in years	62 [IQR 52–71]
Female sex	204 (41%)
Race	
White	302 (60%)
Black	156 (31%)
Asian	11 (2)%
Median Charlson Comorbidity Index	4 [IQR 2–7]
Pre-admission location	
Home	312 (62%)
Skilled nursing facility	79 (16%)
Nursing home	30 (6%)
Transfer from another hospital	81 (16%)
ICU on day of culture collection	297 (59%)
Vasopressor therapy	125 (25%)
Mechanical ventilation	263 (52%)
Severe immune compromise^a^	127 (25%)
Source of isolate	
Pneumonia	307 (61%)
Intra-abdominal fluid	88 (18%)
Bloodstream	30 (6%)
Urinary	77 (15%)

^a^Defined as bone marrow transplant in the previous 12 months, active graft versus host disease on immunosuppressive therapy, chemotherapy in the previous 6 months, solid organ transplant, acquired immunodeficiency syndrome, autoimmune condition requiring steroids or biologics, or an absolute neutrophil count <500 cells/microliter from the time of culture collection until day 7.

AST profiles indicated variable activity across β-lactam agents (Supplemental Excel). Cefepime-zidebactam exhibited the highest activity (100% susceptibility; MIC₅₀/₉₀ 4/8 µg/mL), followed by cefiderocol-xeruborbactam (98%; 0.5/2 µg/mL), cefiderocol (95%; 0.5/2 µg/mL), ceftolozane-tazobactam (90%; 1/4 µg/mL), cefepime-taniborbactam (86%; 4/16 µg/mL), ceftazidime-avibactam (85%; 4/16 µg/mL) and imipenem-relebactam (41%; 0.5/8 µg/mL). Across the cohort, only 178 (35%) isolates were susceptible to all seven agents (Table 2).

**Table 2.** Percent susceptibilities of β-lactam agents against a cohort of 502 *Pseudomonas aeruginosa* isolates exhibiting difficult-to-treat resistance^a^

Antibiotic	Susceptibility breakpoint applied1 (mg/L)	% Susceptible	MIC₉₀ (mg/L)	MIC50 (mg/L)
Cefiderocol	≤4	95%	2	0.25
Ceftazidime-avibactam	≤8/4	85%	16	4
Ceftolozane-tazobactam	≤4/4	90%	4	1
Imipenem-relebactam	≤2/4	41%	8	0.5
Cefepime-taniborbactam	≤8/4	86%	16	4
Cefiderocol-xeruborbactam	≤4/4	98%	2	0.25
Cefepime-zidebactam	≤64/64	100%	8	4

^a^The following interpretive criteria were applied to determine susceptibility: cefepime-taniborbactam ≤8/4 mg/L (Clinical and Laboratory Standards Institute [CLSI] cefepime breakpoint), cefepime-zidebactam ≤64/64 mg/L (CLSI investigational breakpoint), cefiderocol ≤4 mg/L (CLSI breakpoint), cefiderocol-xeruborbactam ≤4/4 mg/L (CLSI cefiderocol breakpoint), ceftazidime-avibactam ≤8/4 mg/L (CLSI breakpoint), ceftolozane-tazobactam ≤4/4 mg/L (CLSI breakpoint), imipenem-relebactam ≤2/4 mg/L (CLSI breakpoint).

Genomic characterization identified carbapenemase genes in a minority of isolates (21, 4%), including *bla*_GES_ (13/21, 2.6%), *bla*_KPC_ (1/21, 0.2%), *bla*_VIM_ (7/21, 1.4%) and *bla*_IMP_ (1/21, 0.2%). A total of 22 carbapenemase genes were identified across the 21 carbapenemase-producing isolates; one isolate co-harboured *bla*_VIM-80_ and *bla*_GES-9_. Only 3 (15%) of 21 patients harbouring carbapenemase-producing *P. aeruginosa* isolates reported travel outside of the USA within the preceding 12 months, based on standardized medical intake documentation that occurred at all participating sites. These included isolates producing IMP (*n* = 1 [Panama]) and VIM (*n* = 2 [Kuwait, India]). No isolates contained *bla*_CTX-M_, *bla*_PER_, or *bla*_VEB_ genes.

Among the 21 carbapenemase-producing isolates, susceptibility was relatively high for the investigational BL-BLI combinations cefiderocol-xeruborbactam (18/21, 86%) and cefepime-taniborbactam (17/21, 81%). In contrast, aside from cefepime-zidebactam (21/21, 100%) and cefiderocol (16/21, 76%), current FDA-approved agents demonstrated limited activity against carbapenemase-producers, including ceftazidime-avibactam (5/21, 24%), imipenem-relebactam (1/21, 5%) and ceftolozane-tazobactam (0/21, 0%). Notable limitations among investigational agents included reduced activity of cefiderocol-xeruborbactam against GES-producing isolates (10/13, 77%), lack of activity of taniborbactam against the single IMP-producing isolate (0/1, 0%) and slightly reduced activity of taniborbactam against GES (11/13, 85%) and VIM-producing isolates (6/7, 86%); Table 3.

**Table 3.** Percent susceptibilities of β-lactam agents against a cohort of 502 *Pseudomonas aeruginosa* isolates exhibiting difficult-to-treat resistance, according to the presence of carbapenemase genes^a^

Agent	*bla*_GES_ (*n* = 13)	*bla*_KPC_ (*n* = 1)	*bla*_VIM_ (*n* = 7)	*bla*_IMP_ (*n* = 1)
Cefiderocol	10 (77%)	1 (100%)	5 (71%)	1 (100%)
Ceftazidime-avibactam	1 (8%)	1 (100%)	3 (43%)	0 (0%)
Ceftolozane-tazobactam	0 (0%)	0 (0%)	0 (0%)	0 (0%)
Imipenem-relebactam	1 (8%)	0 (0%)	0 (0%)	0 (0%)
Cefepime-taniborbactam	11 (85%)	1 (100%)	6 (86)%	0 (0%)
Cefiderocol-xeruborbactam	10 (77%)	1 (100%)	7 (100%)	1 (100%)
Cefepime-zidebactam	13 (100%)	1 (100%)	7 (100%)	1 (100%)

^a^Twenty-two carbapenemase genes identified in 21 isolates. Specific alleles as follows: *bla*_GES-5_ (5), *bla*_GES-7_ (7), *bla*_GES-9_ (1), *bla*_KPC-5_ (1), *bla*_VIM-1_ (1), *bla*_VIM-2_ (5), *bla*_VIM-80_ (1) and *bla*_IMP-18_ (1).

## Discussion

Currently FDA-approved β-lactam agents—namely cefepime-zidebactam, cefiderocol, ceftazidime-avibactam and ceftolozane-tazobactam—demonstrated relatively high activity against a cohort of 502 DTR *P. aeruginosa* clinical isolates collected from the mid-Atlantic United States, whereas susceptibility to imipenem-relebactam was notably lower at 41%. Among carbapenemase-producing isolates, susceptibility declined substantially across all agents, with only cefepime-zidebactam, cefiderocol and investigational BL-BLI combinations retaining activity against the majority of isolates. This variability in activity underscores the importance of evaluating multiple agents to identify effective therapeutic options when DTR *P. aeruginosa* is recovered from clinical specimens.

Carbapenemase-producing *P. aeruginosa* isolates comprised just 4% of the cohort but are likely to increase in prevalence. VIM-producing *P. aeruginosa* are already endemic in parts of Southern and Eastern Europe and Latin America.^8–10^ Longitudinal data from Greece demonstrate an increase in the prevalence of VIM-producing *P. aeruginosa* from approximately 15% in 2011 to over 50% in 2020 among carbapenem-resistant *P. aeruginosa* isolates.^8,10,11^ The USA has started to experience dissemination of VIM-producing *P. aeruginosa*, highlighted by a 2022–2023 multistate outbreak linked to contaminated artificial tears that resulted in eighty-one cases, including 17% with permanent vision loss and a 5% case-fatality rate.^12^

GES enzymes were the most common carbapenemases identified in our cohort. Interestingly, no patients with GES-producing *P. aeruginosa* infections reported international travel within the previous 12 months. While early GES variants (e.g. GES-1) function as extended-spectrum β-lactamases, subsequent mutations—particularly within the Ω-loop—confer carbapenemase activity (e.g. GES-5).^13^ These enzymes exhibit inconsistent inhibition by most available β-lactamase inhibitors (e.g. tazobactam, relebactam), complicating therapeutic selection.^7^ This challenge is further exacerbated by the absence of FDA-cleared diagnostic assays that include *bla*_GES_ as a target. At present, identification of *bla*_GES_ in clinical isolates depends on whole-genome sequencing, which remains impractical for routine clinical use in most settings. Until routine diagnostic platforms incorporate *bla*_GES_ detection, clinicians are forced to rely on phenotypic susceptibility results to inform antibiotic selection, likely delaying initiation of optimal therapy.

The development of newer BL-BLI combinations has been driven by the need to overcome both enzymatic and non-enzymatic resistance mechanisms in *P. aeruginosa*. Taniborbactam is a cyclic boronic acid inhibitor with broad-spectrum activity against serine β-lactamases, as well as select metallo-β-lactamases, through reversible covalent interactions with catalytic residues and zinc coordination within the active site.^14^ Due to electrostatic incompatibilities, it is limited in its ability to inhibit IMP-producing isolates, which fortunately were rare in both our cohort and many regions of the world. Zidebactam, in contrast, functions as a β-lactam ‘enhancer’ with high-affinity binding to PBP2, complementing the activity of its partner β-lactam cefepime, which primarily targets PBP3, thereby exerting a dual mechanism that is partially independent of β-lactamase inhibition, enabling robust activity against both serine and metallo-β-lactamases.^15^ Xeruborbactam is a cyclic boronate inhibitor also with potent activity against both serine and metallo-β-lactamases, offering increased protection to cefiderocol, which has increasingly demonstrated non-susceptibility to DTR *P. aeruginosa*, in part, due to increased enzymatic expression.^16^ Cefiderocol susceptibility was relatively high in our cohort, likely reflecting the low prevalence of carbapenemase-producing isolates. As a result, the addition of xeruborbactam conferred limited incremental benefit. However, its utility in restoring cefiderocol activity is anticipated to be greater in environments with a higher prevalence of carbapenemase-producing *P. aeruginosa*, particularly in regions with dual carbapenemase production.^17^

Several limitations with this work warrant consideration. First, the cohort is geographically restricted and is only generalizable to other regions with a low prevalence of carbapenemase-producing *P. aeruginosa*. Second, although investigational agents demonstrated encouraging *in vitro* activity, the durability of this activity remains uncertain and will require ongoing surveillance as these agents enter clinical use, similar to patterns observed with currently approved BL–BLI combinations. Third, high *in vitro* susceptibility does not necessarily translate to high clinical efficacy. Cefiderocol highlights this complexity; despite potent activity against DTR *P. aeruginosa*, outcomes in randomized trials have been comparable to last-resort agents such as polymyxins.^18–20^ Fourth, iron-depleted CA-MHB was used for susceptibility testing of both cefiderocol and cefiderocol-xeruborbactam. Although the reference testing standard for cefiderocol-xeruborbactam has not been finalized, it is possible that susceptibility testing for this agent may ultimately not require iron depletion. If so, this could be relevant, as preliminary data suggest that iron-depleted media yield lower cefiderocol-xeruborbactam MICs than standard media. The extent to which this methodological distinction may have influenced the cefiderocol-xeruborbactam MICs observed in our cohort remains uncertain. Finally, the FDA has set the cefepime-zidebactam breakpoint at 8/8 mg/L, based on the cefepime breakpoint for *P. aeruginosa*. The CLSI cefepime-zidebactam investigational breakpoint of 64/64 mg/L will be re-reviewed by the CLSI and if it is reduced, the proportion of DTR *P. aeruginosa* isolates in our cohort susceptible to cefepime-zidebactam may decline.

In summary, several currently available agents retain activity against non–carbapenemase-producing *P. aeruginosa*, while investigational agents demonstrate promising activity against both carbapenemase- and non-carbapenemase-producing DTR isolates. Given the notable genomic plasticity of *P. aeruginosa*, sustained activity will depend on judicious use.

## Supplementary Material

dlag138_Supplementary_Data

## Transparency declarations

R.K.S. has received investigator-initiated research funds from AbbVie, Innoviva, Melinta, Merck and Shionogi, and has served as a consultant or on advisory boards for AbbVie, bioMérieux, Entasis, GlaxoSmithKline, Informuta, Melinta, Merck, Qpex and Shionogi. J.M.M. has received investigator initiated unrestricted research grants from Pfizer and MSD. S.E.C. reports receiving personal fees from Philips and Danaher. P.J.S. reports personal fees from bioMérieux, Day Zero Diagnostics, Next Gen Diagnostics, Cepheid, Qpex, Shionogi Inc and GeneCapture. None of the authors with disclosures have disclosures related to the present work. All other authors report no disclosures.

## Supplementary data

Supplemental Excel are available as Supplementary data at *JAC-AMR* Online.
